# Supplementary material for: PEZO-1 and TRP-4 mechanosensors are involved in mating behavior in Caenorhabditis elegans
Source: PNAS Nexus. 2022 Sep 27;1(5):pgac213. doi: 10.1093/pnasnexus/pgac213 (PMC9802279; doi:10.1093/pnasnexus/pgac213)
Supplement: pgac213_Supplemental_Files [file pgac213_supplemental_files.zip › PNASNEXUS-PNASNEXUS-2022-00001-T-s01.docx]

**Supplemental Figure Legends**

**Figure S1. Body size measurements of the wild type and mutant *C. elegans* males.** Sagittal area of the wild type and mutant *C. elegans* males. t-test, the significance threshold was set to 0.05.

**Figure S2. Brood size reduction in *pezo-1(sy1113)* and *pezo-1(av240)* mutants.** In control animals, brood size over the course of three days was normal at around 250 progeny spanning that period. For mutant animals, both *pezo-1(sy1113)* and *pezo-1(av240)* exhibited significantly reduced brood sizes. However, the brood size for *pezo-1(sy1113)* was on average around 80 animals while the brood sizes for *pezo-1(av240)* was on average around 20 animals. Thus *pezo-1(av240)* has a much stronger brood size reduction than *pezo-1(sy1113)*. These results are consistent with previous results by Bai et al^26^.
