# Supplementary figures and images for: PEZO-1 and TRP-4 mechanosensors are involved in mating behavior in Caenorhabditis elegans
Source: PNAS Nexus. 2022 Sep 27;1(5):pgac213. doi: 10.1093/pnasnexus/pgac213 (PMC9802279; doi:10.1093/pnasnexus/pgac213)

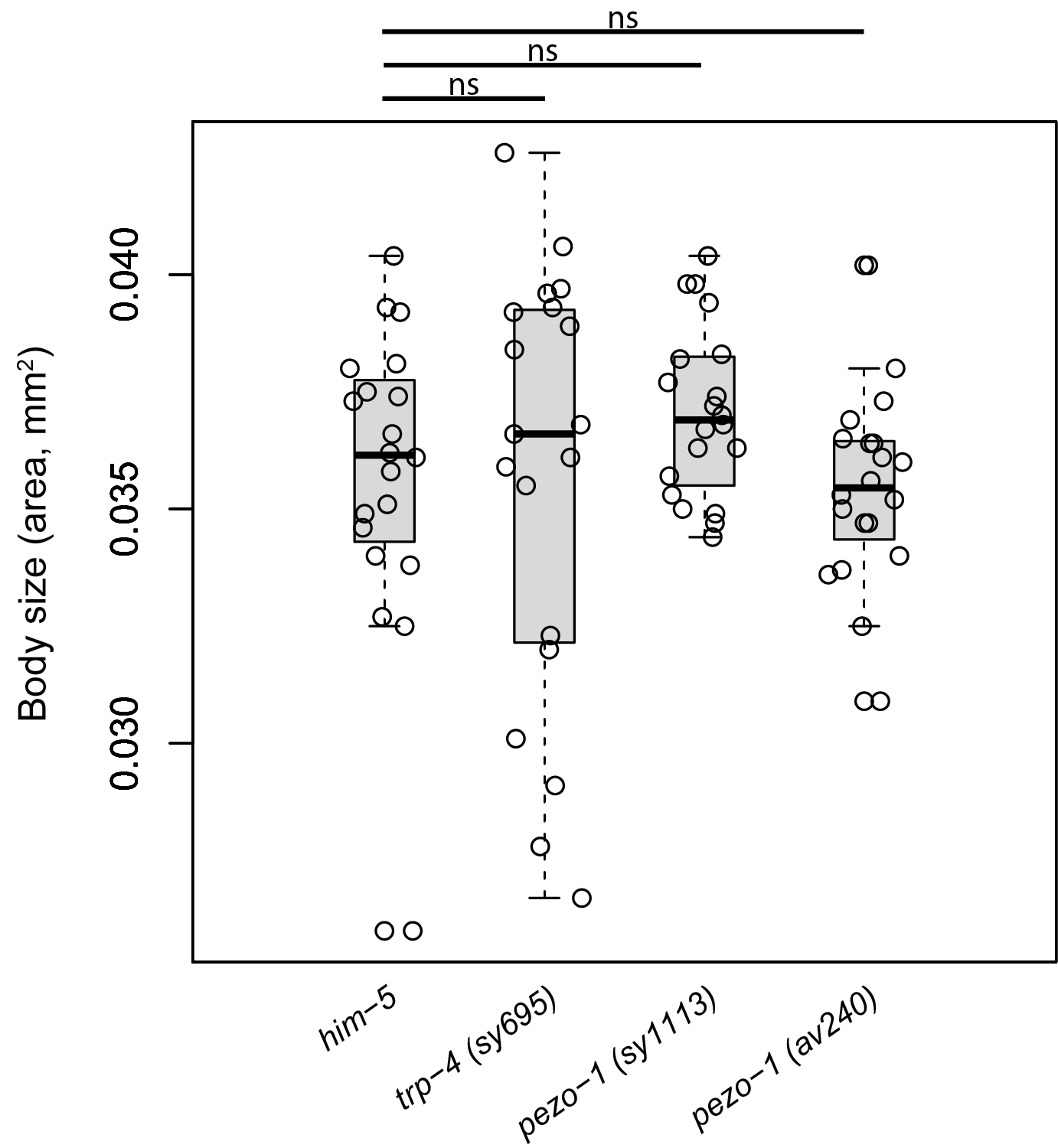

Supplement: pgac213_Supplemental_Files [file pgac213_supplemental_files.zip › PNASNEXUS-PNASNEXUS-2022-00001-T-s02.tif]

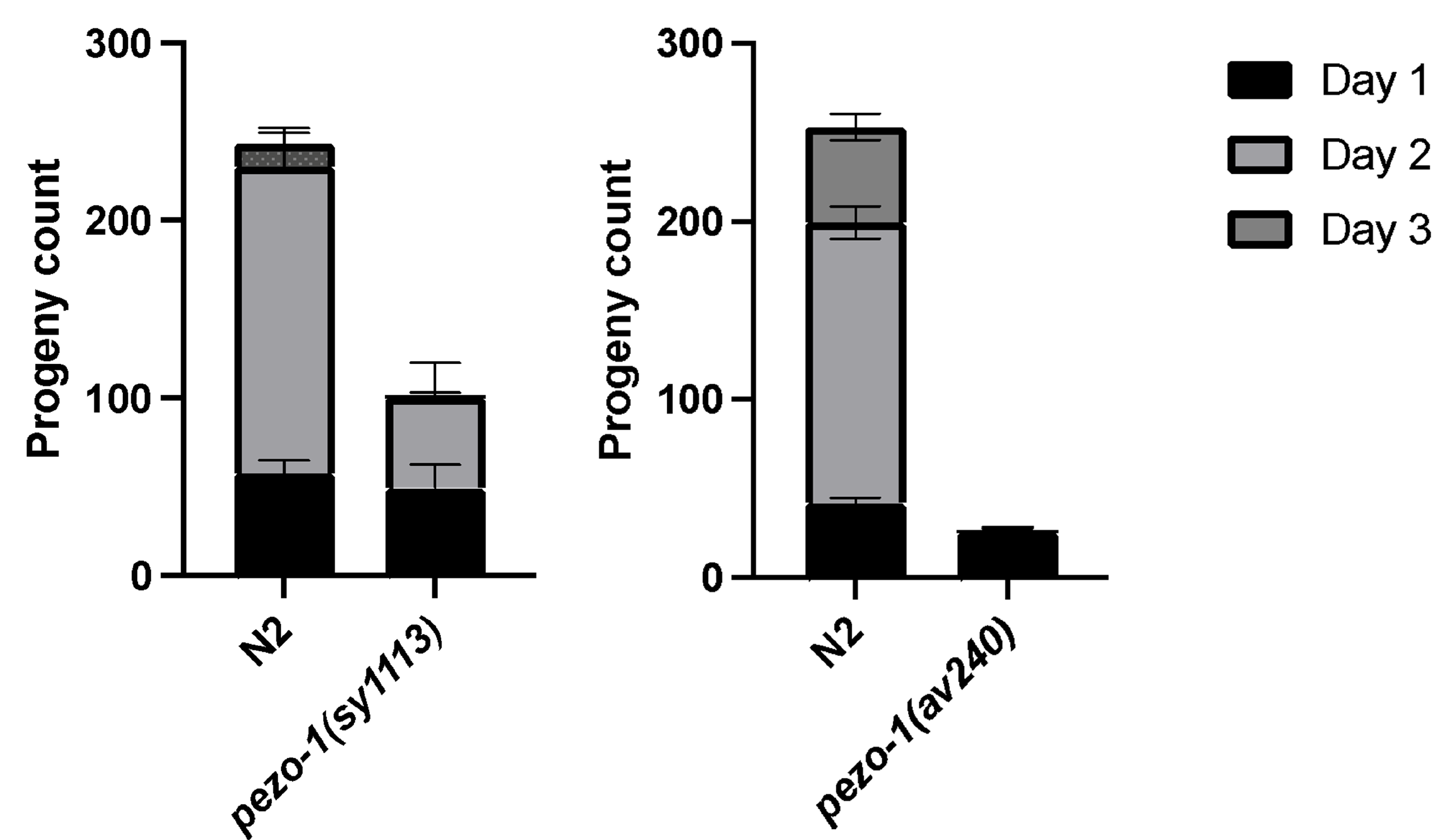

Supplement: pgac213_Supplemental_Files [file pgac213_supplemental_files.zip › PNASNEXUS-PNASNEXUS-2022-00001-T-s03.tif]
